# Vinorelbine plus 3-weekly trastuzumab in metastatic breast cancer: a single-centre phase 2 trial

**DOI:** 10.1186/1471-2407-7-50

**Published:** 2007-03-20

**Authors:** Ermelinda De Maio, Carmen Pacilio, Adriano Gravina, Alessandro Morabito, Francesca Di Rella, Vincenzo Labonia, Gabriella Landi, Francesco Nuzzo, Emanuela Rossi, Pasqualina Silvestro, Gerardo Botti, Maurizio Di Bonito, Maria Pia Curcio, Franca Formichelli, Franca La Vecchia, Maria Staiano, Nicola Maurea, Giuseppe D'Aiuto, Massimiliano D'Aiuto, Renato Thomas, Giuseppe Signoriello, Francesco Perrone, Andrea de Matteis

**Affiliations:** 1 Clinical Trials Unit, National Cancer Institute, Via M. Semmola, I-80131 Naples, Italy; 2 Medical Oncology, National Cancer Institute, Via M. Semmola, I-80131 Naples, Italy; 3 Pathology, National Cancer Institute, Via M. Semmola, I-80131 Naples, Italy; 4 Cardiology, National Cancer Institute, Via M. Semmola, I-80131 Naples, Italy; 5 Senology, National Cancer Institute, Via M. Semmola, I-80131 Naples, Italy; 6 Medical Statistics Unit, Department of Medicine and Public Heath, Second University; Via L. Armanni 5, I-80137 Naples, Italy

## Abstract

**Background:**

After two studies reporting response rates higher than 70% in HER2-positive metastatic breast cancer with weekly trastuzumab and vinorelbine, we planned a phase 2 study to test activity of the same combination, with trastuzumab given every 3 weeks.

**Methods:**

Patients with HER2-positive metastatic breast cancer (3+ at immunohistochemistry or positive at fluorescence in situ hybridization), PS ≤2, normal left-ventricular ejection fraction (LVEF) and no more than one chemotherapy line for metastatic disease were eligible. Vinorelbine (30 mg/m^2^) was given on days 1&8 every 21 and trastuzumab (8 mg/kg day 1, then 6 mg/kg) every 21 days). A single-stage phase 2 design, with p_0 _= 0.45, p_1 _= 0.65, type I and II error = 0.10, was applied; 22 objective responses were required in 39 patients.

**Results:**

From Nov 2002 to May 2005, 50 patients were enrolled, with a median age of 54 years (range 31–81). Among 40 patients eligible for response assessment, there were 7 complete and 13 partial responses (overall response rate 50%; 95% exact CI 33.8–66.2); 11 patients had disease stabilization, lasting more than 6 months in 10 cases. Response rate did not vary according to patients and tumor characteristics, type and amount of previous chemotherapy. Within the whole series, median progression-free survival was 9.6 months (95% CI 7.3–12.3), median overall survival 22.7 months (95% CI 19.5-NA). Fifteen patients (30%) developed brain metastases at a median time of 12 months (range 1–25). There was one toxic death due to renal failure in a patient receiving concomitant pamidronate. Twenty-three patients (46%) had grade 3–4 neutropenia, 2 (4%) grade 3 anemia, 4 (8%) febrile neutropenia. Two patients stopped treatment because of grade 2 decline of LVEF and one patient because of grade 2 liver toxicity concomitant with a grade 1 decline of LVEF. One patient stopped trastuzumab after 50 cycles because of grade 1 decline of LVEF.

**Conclusion:**

Although lower than in initial studies, activity of 3-weekly trastuzumab plus vinorelbine fell within the range of results reported with weekly schedules. Toxicity was prevalently manageable. This combination is safe and active for metastatic breast cancer patients who received adjuvant taxanes with anthracyclines.

## Background

Metastatic breast cancer is an incurable disease, frequently treated with chemotherapy, particularly when hormonal treatment has failed or is not indicated because of lack of estrogen receptor expression in the tumor.

One of the major advances for the treatment of metastatic breast cancer has been the introduction of trastuzumab, a monoclonal antibody directed against the HER2 extracellular domain. Tumors with HER2 overexpression or amplification account for 25–30% of breast cancer and are a particular subgroup with different prognosis, natural history and drug sensitivity [1,2]. The combination of trastuzumab with chemotherapy has been shown to be effective in the treatment of metastatic breast cancer patients in a pivotal study, where the monoclonal antibody was combined with either adriamycin/cyclophosphamide or paclitaxel [3]; significant advantages were reported for all the efficacy end-points, including quality of life that has been reported more recently [4].

Following this report, many studies have been conducted combining trastuzumab with other chemotherapeutic agents frequently used in breast cancer treatment. Preclinical studies have demonstrated that synergistic effects can be obtained with the combination of trastuzumab with vinorelbine or taxanes [5,6].

When we planned the present study, there were reports on two phase 2 studies of the combination of trastuzumab and vinorelbine [7,8]. Both studies employed the weekly schedule of trastuzumab and produced exciting results with very high response rates even in patients receiving it as second-line treatment.

Based on such data, we planned a single-centre phase 2 study to test activity and tolerability of the same combination of trastuzumab and vinorelbine, with trastuzumab given 3-weekly, as first- or second-line treatment of metastatic breast cancer patients overexpressing HER2. The 3-weekly schedule of trastuzumab had been shown to have similar pharmacokinetic profile and activity to weekly schedule in a previous phase two study [9] but could be more convenient for patients, and potentially less costly.

## Methods

### Eligibility criteria

Patients were eligible if they had histologically proven, metastatic breast cancer and overexpression of HER2 defined as score 3+ by immunohistochemistry (IHC) or as gene amplification by fluorescence in-situ hybridization (FISH). They could have received no more than one chemotherapy line for metastatic disease (also neo-adjuvant and/or adjuvant chemotherapy were allowed). Other eligibility criteria included: performance status ≤2 according to the Eastern Cooperative Oncology group (ECOG) scale, adequate bone marrow (absolute neutrophil count ≥1500/mm^3^, platelets ≥100000/mm^3 ^and hemoglobin ≥8 g/dl), renal (serum creatinine ≤1.5 × upper normal limit) and hepatic (SGOT and SGPT ≤2.5 × upper normal limit and bilirubin ≤1.5 × upper normal limit, unless due to liver metastases) function. Patients were excluded if they had left ventricular ejection fraction (LVEF) <50% as determined by echocardiography, symptomatic brain metastases, a positive history of other types of cancer (except for radically resected carcinoma-in-situ of the cervix or non-melanoma skin cancer), or previous treatment with trastuzumab or vinorelbine. Patients with no target lesions were eligible for toxicity, but not for activity assessment. The study was approved by the Institutional Ethical Committee and all patients provided written informed consent before starting study procedures.

### Treatment plan, dose modifications and delay

For the first cycle, patients received trastuzumab on day 1 at loading dose of 8 mg/kg in a 90-minute infusion and vinorelbine on days 2 and 8 at 30 mg/m^2^. For the subsequent every 3 week cycles, trastuzumab was given at dose of 6 mg/kg on day 1 and vinorelbine was given on days 1 and 8. Trastuzumab was administered as a 90-minute infusion prior to vinorelbine for all cycles following the initial dose. Vinorelbine was administered for a maximum of 9 cycles, while trastuzumab was continued until disease progression or unacceptable toxicity.

Dose adjustments were planned only for vinorelbine and were dependent on the type and grade of toxicity observed: vinorelbine dose was reduced by 20% for neutrophil count between 1000 and 1499/mm^3 ^or platelet count between 75000 and 99000/mm^3^. In case of neutrophil count <1000/mm^3 ^or platelets <75000/mm^3^, vinorelbine was delayed for one week (day 1) or omitted (day 8). Patients with treatment-related grade ≥2 non-hematologic toxicity (except LVEF alterations) did not receive vinorelbine and trastuzumab therapy until the toxicity resolved to grade 1 or lower. If toxicity failed to resolve after a 2-week treatment delay, patients were taken off protocol.

In case of reduction of LVEF ≤44%, treatment with trastuzumab was stopped and LVEF measurements were repeated after 3 weeks; if LVEF was confirmed ≤44% or <50% with a decrease ≥10 points as compared to baseline, trastuzumab treatment was definitively stopped. If LVEF was ≥50% or >44% with a decrease <10 points as compared to baseline, trastuzumab was resumed with an intensive cardiac surveillance.

Hematologic support with G-CSF and recombinant human erythropoietin was used according to standard guidelines.

### Pre-treatment procedures

Pre-treatment staging was performed within one month before starting therapy and included a complete history and physical examination, complete blood cell counts and serum chemistries, ECG, LVEF evaluated by echocardiography with biplane method according to Simpson's rule, chest X-ray, complete abdominal ultrasound, bone scan. Other exams were performed if clinically indicated. To define dominant metastatic site, visceral sites (lung, liver, brain, pleura) prevailed over all other sites, and bone prevailed over soft tissues (breast, nodes, skin).

The HER2 status was assessed on primary breast tumor after resection or on core biopsy, by immunohistochemistry (IHC) and/or fluorescence in situ hybridization (FISH) on formalin-fixed paraffin-embedded tissue according to the guidelines reported in 2000 by Ellis et al [10]. The level of staining of non-neoplastic epithelium present on the same slide was taken into consideration.

IHC was performed using DAKO HercepTest approved in 1998 by the Food and Drug Administration. Scores of 2+ and 3+ were considered positive (overexpression). HER-2/neu gene amplification by FISH was performed for all cases of 2+ score. FISH analysis was performed using the Vysis PathVysion HER2 probe kit according to manufacturer's instructions. Two pathologists evaluated FISH and IHC analyses independently.

### Evaluation of response and toxicity

Response assessment was planned after the completion of the 3^rd ^and 6^th ^cycles of chemotherapy, repeating the staging procedures. Response was evaluated according to RECIST (Response Evaluation Criteria in Solid Tumor) guidelines [11]. Tumor lesions were categorized as measurable if they could be accurately measured in at least one dimension as ≥20 mm with conventional techniques or as ≥10 mm with spiral CT. All other tumor lesions, including small lesions and truly non-measurable lesions, were categorized as non-target lesions.

Physical examination and vital signs were recorded before each cycle of chemotherapy. Complete blood cell count was measured before every cycle and weekly, biochemistry and urine analysis before day 1 of each cycle. Cardiac assessment was performed by clinical evaluation, electrocardiography and echocardiography with LVEF measurements every 3 cycles or when clinically indicated. Toxicity was graded according to National Cancer Institute Common Toxicity Criteria (NCI CTC), version 2.0.

### Study design and sample size

A single-stage, phase 2 study design was applied. The primary end-point was response rate. With minimum acceptable proportion of response (P_0_) of 0.45, desired proportion of response (P_1_) of 0.65, α error 0.10, β error 0.10, 39 patients assessable for response were needed. For defining the study as positive, at least 22 objective responses should be recorded. Exact 95% confidence interval (CI) were calculated. Secondary end-points were toxicity, progression-free survival, defined as the time from the date of enrolment and the date of the first progression, and overall survival, defined as the time between the date of study enrolment and the date of death. Progression-free and overall survival were estimated using the Kaplan-Meier method [12].

## Results

### Patients characteristics

From November 4^th^, 2002 to May 11^th^, 2005, 50 patients entered the study, of whom 40 were eligible for response assessment. Main baseline characteristics of the patients are reported in Table 1. Median age was 54 years (range 31–81); one-fifth of patients were older than 65. ECOG performance status was 0 in 41 patients, 1 in 7 patients and 2 in 2 patients. Nine patients, who were ineligible for other standard treatments because of advanced age or concomitant cardiologic disease, were chemo-naive; among the remaining 41 patients, 37 had received previous anthracyclines and 13 had received taxanes. Overall, considering neoadjuvant, adjuvant and metastatic settings, 26 patients had received one prior course of chemotherapy and 15 had received two courses. No patient had received prior trastuzumab or vinorelbine. More than half of the patients had metastatic disease to a single organ. The predominant metastatic sites were visceral, and 8 patients had bone metastases combined with visceral sites.

### Compliance and toxicity

A total number of 315 cycles of chemotherapy (median number of 9 cycles per patient, range 1 to 9) and of 487 administrations of trastuzumab (median number of 9 administrations per patient, range 1–50) were given to the 50 patients. Reasons for treatment discontinuation were one toxic death, one early death due to progression of disease, 8 non fatal toxicity, 10 progression of disease and one patient refusal. G-CSF support was required in 72 cycles of chemotherapy (22.6%). The planned dose intensity of vinorelbine was 20 mg/m^2^/wk and the median dose actually delivered was 15.7 mg/m^2^/wk; therefore the relative dose intensity was 0.79. The planned dose intensity of trastuzumab was 2 mg/kg/wk and the median dose actually delivered was 1.84 mg/kg/wk with a relative dose intensity of 0.9.

Hematological and non-hematological toxicities are reported in table 2.

Grade 3–4 neutropenia occurred in almost the half of patients (46%) and 4 patients developed febrile neutropenia. Grade 4 anemia and thrombocytopenia were never observed.

The most common non-hematological toxicities were fatigue, constipation and abdominal pain. A toxic death was observed after the first cycle of chemotherapy, due to acute renal failure in a patient with bone metastases who was concomitantly treated with pamidronate. One patient without liver metastases developed grade 3 liver toxicity after the fifth cycle of chemotherapy due to a re-exacerbation of HCV-related hepatitis.

Patients were strictly monitored with frequent evaluation of LVEF and a treatment related cardiac event was recognized in four patients. During the period of combined treatment there were three cases of LVEF decline. The first patient presented with tachycardia and an echocardiogram showed a reduction of resting LVEF >20% from baseline value (grade 2 according to NCI-CTC) after the third cycle; she had hypertension and retro-sternal goitre with hyperthyroidism as comorbidity and had received adjuvant epirubicin (total dose 480 mg/m^2^); the LVEF reduction was persistent for at least 9 months after treatment. The second event was an asymptomatic grade 2 decline of LVEF after the third cycle, that was persistent three weeks after interruption of treatment; this patient, who had been treated with adjuvant epirubicin for a total dose of 400 mg/m^2^, developed brain metastases and died four months later. The third cardiac event was a grade 1 asymptomatic decline of LVEF after three cycles, in a patient who had not received previous anthracyclines; this patient was taken off from the study because of concomitant grade 2 liver toxicity. One more event of grade 1 LVEF decline was reported in a patient after 50 overall cycles of trastuzumab, with LVEF declining from 80%, at baseline, to 66%.

### Activity

Among 40 eligible patients, a complete response was observed in 7 (17.5%) and a partial response in 13 (32.5%) cases, for an overall response rate of 50% (95% exact CI 33.8–66.2). Median duration of response among patients treated as first-line was 12 months (range 5–27). Disease stabilization was obtained in 11 patients, lasting more than 6 months in 10 of them (range 3–13).

Ten patients were not eligible for response assessment because of the absence of target lesions; according to the outcome of non-target lesions, 4 complete responses (lasting 7, 12, 12 and 43 months), 2 non response/non progression (lasting 8 and 12 months) and 3 progressions were reported, while one patient was not reassessed because of unacceptable toxicity (febrile neutropenia and abdominal pain) before the third cycle administration. Interestingly, one patient who achieved a complete response continued trastuzumab until 50 administrations; treatment was then interrupted due to an asymptomatic reduction of LVEF from 80% to 66%.

In table 3, the rate of responding patients in the whole series is scattered by main baseline characteristics of patients, tumor and pretreatment that could potentially affect the chance of response. Confidence intervals suggests that none of the considered variables significantly affected the chance of response. However, with limitations due to the small sample size, it appears that elderly patients, those with a poorer performance status and those with a longer time from breast cancer onset had lower response rates.

Brain was the first site of progression in 9 cases, but, overall 15 patients (30%) developed brain metastases, at times ranging from 1 to 25 months (median 12 months).

**Figure 1:**
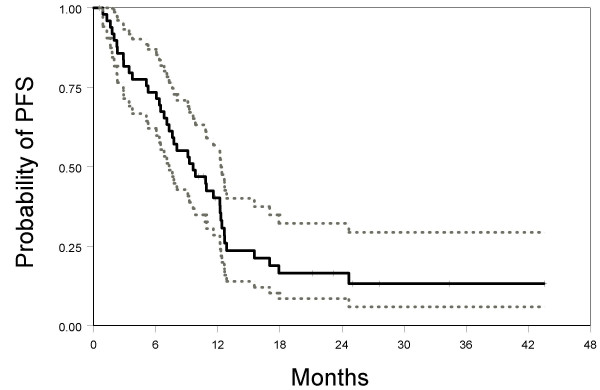
Kaplan-Meier estimated progression-free survival curve (solid line) with 95% confidence limits (dotted lines).

**Figure 2:**
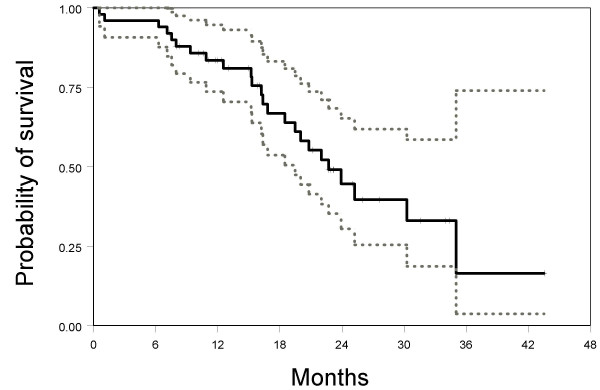
Kaplan-Meier estimated overall survival curve (solid line) with 95% confidence limits (dotted lines).

Up to July 1^st ^2006, 40 patients had suffered progression of disease and 24 patients had died. Median progression-free survival was 9.6 months (95% CI: 7.3–12.3). Median overall survival was 22.7 months (95% CI: 19.5-NA). Kaplan-Meier's estimated curves of progression-free and overall survival are reported in Figures 1 and 2.

## Discussion

In the present study we tested the combination of trastuzumab plus vinorelbine in the treatment of HER-2 positive metastatic breast cancer. The response rate we found was lower than in the initial phase 2 studies of this combination [7,8] but nevertheless compatible with a positive interpretation, given that in the near future the proportion of metastatic breast cancer patients who are eligible for a treatment with trastuzumab, but have already received taxanes as adjuvant, will increase.

Chan recently reviewed literature available on the combination of trastuzumab with vinorelbine [13]. She found eleven trials of the weekly schedule, but the present one (reported in a preliminary form to the National Italian Congress in 2004) was the only one testing a 3-weekly schedule of trastuzumab. Among the eleven studies of the weekly schedule, response rate ranged from 44% to 86%; complete response rate ranged from 3% to 15%; grade 3–4 neutropenia was reported in 23% to 84% of patients; febrile neutropenia in less than 5% of patients. Our results compare well with such data; the overall response rate falls within the range of previously reported studies, although at the lower end of the values; the complete response rate is higher than previous data; neutropenia incidence is in the midrange of previous results; febrile neutropenia has been more frequent in our study.

While the administration of vinorelbine on days 1 and 8 every 3 weeks is a commonly used scheme, the use of trastuzumab every 3 weeks is innovative in combination with vinorelbine. Only one publication has recently focused on a similar schedule; Bartsch et al [14] reported a chart review showing that out of 17 patients, treated with oral vinorelbine on days 1 and 8 combined with 3-weekly trastuzumab, 9 had an objective response that was complete in 4 cases (23.5%). However, the retrospective nature and the small number of patients require caution in the interpretation of these data. The schedule that we tested differs from previous studies in several aspects. Two studies have been reported addressing pharmacokinetics (PK), safety and activity of the 3-weekly trastuzumab that we used. In the first one [9], trastuzumab was combined with paclitaxel in 36 patients; PK data showed that plasma levels for the drug were sufficiently high at the end of cycle 1 and suggested that half-life of trastuzumab combined with paclitaxel might be 18 to 27 days, encouraging the use of the 3-weekly schedule; one-third of patients had a >15% decline of LVEF, but only one developed symptomatic heart failure. The second paper [15] reported data on trastuzumab given as single agent. In this study, with 105 patients, there was no significant change of LVEF, and PK data confirmed that that the 3-weekly schedule produced an exposure to the drug similar to the weekly one.

Several retrospective reports in the last years suggested an increased prevalence of brain metastases in patients receiving trastuzumab-based therapy for HER2-overexpressing metastatic breast cancer [16-19]. The true incidence of brain metastases in breast cancer is not precisely known, but it's estimated close to 10–15% of patients even if autopsy series reported rates ranging from 18% to 30% [19]. In our study, the rate of patients who developed brain metastases was quite high (30%). Our interpretation is that this only represents an apparent increase of incidence, probably due to the durable effect of trastuzumab on the other metastatic sites; similar data, indeed, have been also reported with the introduction of other highly effective chemotherapies, like platinum-based for ovarian cancer [20] and taxane-based for breast cancer [21].

Following the pivotal trial of Slamon et al. [3] and several other reports, including one recent by Marty et al. testing the combination of trastuzumab with docetaxel [22], trastuzumab is actually indicated for clinical practice in combination with taxanes. However, such combinations would not be reasonable for the patients who have already received taxanes as adjuvant treatment, a group whose number is going to increase. It must also be considered that the use of trastuzumab as adjuvant treatment, prompted by excellent results of the first trials [23,24], will probably reduce the need of treating metastatic breast cancer patients with trastuzumab, and other molecules, like lapatinib [25] or others at a less advanced stage of development, will be tested. In the meantime, we believe that the combination of trastuzumab and vinorelbine can be a rationale and safe approach for the treatment of HER-2 positive metastatic breast cancer patients who have not received trastuzumab in the adjuvant setting. Further improvement of the activity of this combination could derive by the addition of a third drug, like carboplatin that could be synergistic with trastuzumab [26]; a phase 2 trial of this 3-drug combination is currently ongoing in our institution.

## Conclusion

The combination of trastuzumab and vinorelbine is an active and rationale approach in metastatic breast cancer with a manageable toxicity profile, and can be considered for patients with HER2-positive breast cancer who have already received anthracyclines and taxanes.

## Competing interests

The author(s) declare that they have no competing interests.

## Authors' contributions

ADM and FP projected the study. FP and GS were responsible of the statistical design. EDM, CP, AG, FDR, VL, GL, FN, ER, PS, GDA, MDA, RT and ADM enrolled patients, treated them according to study protocol and collected study data. GB, MDB, MPC, FF, FLV and MS were responsible of pathologic assessment and HER2 testing. NM was responsible for cardiologic assessment of patients. EDM, AM, GS, and FP performed the analysis. EDM produced the first draft of the manuscript. All gave substantial intellectual contribution when revising the manuscript. All read and approved the final manuscript.

## Pre-publication history

The pre-publication history for this paper can be accessed here:



## Supplementary Material

Additional file 1 NCI Naples Breast Cancer Group. the names of researchers involved in the present study and in the activities of the NCI Naples Breast Cancer Group Click here for file

## Figures and Tables

**Table 1:** Baseline characteristics of patients (N = 50)

**Variable**		
Age, n (%)		
<65	40	(80)
65 or more	10	(20)
Histotype, n (%)		
Ductal	41	(82)
Lobular	6	(12)
Other	3	(6)
ER status, n (%)		
Negative	19	(38)
Positive	30	(60)
Unknown	1	(2)
Performance status, n (%)		
0	41	(82)
1	7	(14)
2	2	(4)
HER2 status, n (%)		
Score 3+ by IHC	47	(94)
Score 2+ and positive by FISH	3	(6)
Time from breast cancer diagnosis, n (%)		
<2 years	19	(38)
2 years or more	31	(62)
Previous chemotherapy, n (%)		
No	9	(18)
Yes	41	(82)
By type of drugs		
CMF	4	(8)
with anthracycline	24	(48)
with anthracycline and taxane	13	(26)
By number of lines of chemotherapy		
one line	26	(52)
two lines	15	(30)
Extension of disease, n (%)		
By number of involved organs		
1	27	(54)
2	16	(32)
3	7	(14)
By dominant metastatic site		
soft tissues	13	(26)
viscera	37	(74)
Eligible for response assessment, n (%)		
No	10	(20)
Yes	40	(80)

IHC: immunohistochemistry, FISH: fluorescence in situ hybridization

**Table 2:** Worst per-patient toxicity within the period of combined treatment (N = 50)

**Type of toxicity**	**Patients (%)**				
	
	**1**	**2**	**3**	**4**	**5**
Allergy	2 (4)	2 (4)	2 (4)	-	-
Anemia	18 (36)	11 (22)	2 (4)	-	-
Leukopenia	-	11 (22)	7 (14)	1 (2)	-
Neutropenia	-	5 (10)	13 (26)	10 (20)	-
Febrile neutropenia	-	-	4 (8)	-	-
Platelets	1 (2)	2 (4)	-	-	
Cardiac (LVEF decline)	1 (2)	2 (4)	-	-	-
Renal	1 (2)	1 (2)	-	-	1 (2)
Fatigue	12 (22)	4 (8)	-	-	-
Fever	2 (4)	1 (2)	-	-	-
Hair loss	1 (2)	-	-	-	-
Skin	3 (6)	4 (8)	-	-	-
Anorexia	2 (4)	-	-	-	-
Constipation	12 (24)	3 (6)	-	-	-
Diarrhoea	3 (6)	-	-	-	-
Nausea	5 (10)	2 (4)	-	-	-
Stomatitis	3 (6)	-	-	-	-
Vomiting	4 (8)	-	-	-	-
Liver	1 (2)	1 (2)	1 (2)	-	-
Sensorial neuropathy	4 (8)	-	-	-	-
Abdominal pain	13 (26)	1 (2)	1 (2)		
RBC transfusion	-	-	1 (2)	-	-

RBC = red blood cell

**Table 3:** Rate of responding patients according to baseline characteristics

**Variable**	**no. of patients**		**response rate**
		
	**at risk**	**responding**	**(95% exact CI)**
Age			
<65	40	21	52.5 (36.1–68.5)
65 or more	10	3	30.0 (6.7–65.2)
Histotype			
Ductal	41	20	48.8 (32.9–64.9)
Lobular or other	9	4	44.4 (13.7–78.8)
ER status			
Negative	19	9	47.4 (24.5–71.1)
Positive	30	14	46.7 (28.3–65.7)
Performance status			
0	41	22	53.7 (37.4–69.3)
1 or 2	9	2	22.2 (2.8–60.0)
Time from breast cancer diagnosis			
<2 years	19	12	63.2 (38.4–83.7)
2 years or more	31	12	38.7 (21.8–57.8)
Previous chemotherapy			
No	9	4	44.4 (13.7–78.8)
Yes	41	20	48.8 (32.9–64.9)
By type of drugs			
CMF	4	2	50.0 (6.8–93.2)
with anthracycline	24	13	54.2 (32.8–74.5)
with anthracycline and taxane	13	5	38.5 (13.9–68.4)
By number of lines of chemotherapy			
one line	26	13	50.0 (29.9–70.1)
two lines	15	7	46.7 (21.3–73.4)
Extension of disease			
By number of involved organs			
1	27	13	48.1 (28.7–68.0)
2	16	7	43.7 (19.7–70.1)
3	7	4	57.1 (18.4–90.1)
By dominant metastatic site			
soft tissues	13	7	53.8 (25.1–80.8)
viscera	37	17	45.9 (29.5–63.1)
