# Supplementary material for: Vinorelbine plus 3-weekly trastuzumab in metastatic breast cancer: a single-centre phase 2 trial
Source: BMC Cancer. 2007 Mar 20;7:50. doi: 10.1186/1471-2407-7-50 (PMC1832208; doi:10.1186/1471-2407-7-50)
Supplement: Additional file 1 — NCI Naples Breast Cancer Group. the names of researchers involved in the present study and in the activities of the NCI Naples Breast Cancer Group [file 1471-2407-7-50-S1.doc]

**Appendix**

Members of the NCI-Naples Breast Cancer Group who contributed to this manuscript:

- *Cell Biology and Preclinical Models Unit*: Nicola Normanno, Amelia D’Alessio, Monica Rosaria Maiello, Adele Carotenuto, Gianfranco De Feo, Anna Maria Rachiglio.
- *Clinical Trials Unit*: Francesco Perrone, Alessandro Morabito, Ermelinda De Maio, Massimo Di Maio, Mario Iaccarino, Maria Carmela Piccirillo, Antonella De Luca, Roberta D’Aniello, Jane Bryce, Fabiano Falasconi.
- *Medical Oncology C*: Andrea de Matteis, Francesca Di Rella, Adriano Gravina, Vincenzo Labonia, Gabriella Landi, Francesco Nuzzo, Carmen Pacilio, Emanuela Rossi, Rosa Fiore.
- *Nuclear Medicine*: Secondo Lastoria, Corradina Caracò, Luigi Aloj.
- *Pathology*: Gerardo Botti, Maurizio Di Bonito, Maria Pia Curcio, Franca Formichelli, Franca La Vecchia, Maria Staiano.
- *Pharmacy*: Maria Rosaria Salzano, Piera Maiolino.
- *Radiology*: Alfredo Siani, Teresa Petrosino, Rosaria Rubulotta, Paolo Vallone, Mauro Matta Ceraso, Antonella Petrillo, Orlando Catalano, Fabio Sandomenico.
- *Radiotherapy*: Brunello Morrica.
- *Senology*: Giuseppe D’Aiuto, Franca Avino, Immacolata Capasso, Massimiliano D’Aiuto, Claudio Longo, Massimo Rinaldo, Renato Thomas.
- *Medical Statistics (Second University of Naples)*: Ciro Gallo, Giuseppe Signoriello, Paolo Chiodini.
